# A novel PolyAr87-based cell transfection protocol for nanobody expression optimized via a targeted design of transfection approach

**DOI:** 10.3389/fbioe.2025.1720185

**Published:** 2026-03-13

**Authors:** Lautaro Fidel Bracco, Giovanna Lucia Liguori, Antonella Lanati, Juan Manuel Lázaro-Martínez, Mariela Bollini, Leonardo Poggio, Marina Bok, Lorena Itatí Ibañez, Viviana Parreño

**Affiliations:** 1 Centro de Investigación en Ciencias Veterinarias y Agronómicas (CICVYA), INTA, Hurlingham, Buenos Aires, Argentina; 2 Institute of Genetics and Biophysics (IGB) “Adriano Buzzati Traverso”, National Research Council (CNR) of Italy, Naples, Italy; 3 Valore Qualità, Pavia, Italy; 4 Universidad de Buenos Aires, Facultad de Farmacia y Bioquímica, Ciudad autónoma de Buenos Aires, Argentina; 5 CONICET-Universidad de Buenos Aires, Instituto de Química y Metabolismo del Fármaco (IQUIMEFA), Ciudad Autónoma de Buenos Aires, Argentina; 6 Centro de Investigaciones en Bionanociencias (CIBION – CONICET), Polo Científico Tecnológico, Ciudad Autónoma de Buenos Aires, Argentina; 7 Instituto de Virología e Innovación Tecnológica, IVIT, CONICET-INTA, Hurlingham, Buenos Aires, Argentina; 8 Instituto de Química, Física de los Materiales, Medioambiente y Energía (INQUIMAE-UBA-CONICET), Ciudad Autónoma de Buenos Aires, Argentina

**Keywords:** design of experiment (DoE), HEK293, method optimization, PolyAR reagent, transfection, nanobody

## Abstract

**Introduction:**

Transfection is a fundamental technique for introducing foreign nucleic acids into eukaryotic cells, widely used in biotechnology for recombinant protein expression. Nanobodies fused to enzymes are key reagents in the development of diagnostic tests such as ELISA. Here, we optimized a novel, low-cost, ready-to-use linear polyethyleneimine (PEI)-based transfection reagent, PolyAr87, for the efficient delivery of a plasmid encoding a nanobody-HRP fusion protein into HEK293T cells.

**Results:**

When compared with other commonly used transfection reagents—including branched and linear PEI and FuGene® 6—PolyAr87 showed superior performance over PEI powders and comparable efficacy to FuGene® 6 at a substantially lower cost. Then, using a Design of Experiments (DoE) approach, specifically the Design of Transfection (DoT) model, we applied a two-phase optimization strategy comprising a Full Factorial Design (FFD) and a Box–Behnken Design (BBD) to identify and fine-tune key factors affecting transfection efficiency. PolyAr87 concentration and DNA concentration were found to significantly influence outcomes, with optimal efficiency achieved at 1.75 μg/mL of DNA and 5.0 μg/ mL of PolyAr87.

**Discussion:**

Model validation demonstrated strong predictive power and reproducibility. These findings confirm both the effectiveness of PolyAr87 as a cost-efficient transfection reagent and the utility of DoT-based optimization for enhancing gene delivery protocols in mammalian cell systems.

## 1 Introduction

Transfection is the process by which foreign nucleic acids are introduced into eukaryotic cells to generate genetically modified cells. This process is useful for recombinant protein production, as well as for mutational analysis and for modulating gene expression by gain-of-function or loss-of-function experiments (Chong et al., 2021).

Cultivated mammalian cells are the most commonly used system for recombinant protein expression for clinical applications. This platform guarantees proper protein folding and correct post-translational processing, allowing human protein-like molecular structure assembly (Gomes et al., 2016). Therefore, biotherapeutics such as monoclonal antibodies, fusion proteins, and enzymes have been produced to treat various pathologies, such as cancer and metabolic and autoimmune diseases. At present, more than 60% of the therapeutic proteins approved and on the market are produced from mammalian cells (Walsh and Walsh, 2022).

Transfection can be classified into two types, namely, stable and transient (Chong et al., 2021). In stable transfection, the goal is to integrate foreign DNA into the host nuclear genome or to maintain an episomal vector in the host nucleus as an extrachromosomal element (Fus-Kujawa et al., 2021); then, the transgene can be constitutively expressed. The main disadvantage of stable transfection is that it requires selective screening for the isolation of stable transfectants, which requires prolonged periods.

On the other hand, transient transfection does not require integrating nucleic acids into the host cell genome. For this purpose, nucleic acids must be transfected as plasmids or oligonucleotides, with the disadvantage that nucleic acids are lost with subsequent cell replication (Chong et al., 2021).

The choice between transient and stable transfection usually depends on the experimental goals and cell type. Transient transfection is often used for studying the effects of short-term expression of genes or gene products, such as gene knockdown or silencing with inhibitory RNAs (Fus-Kujawa et al., 2021). Additionally, for rapid recombinant protein production in small- to medium-sized volumes, transient transfection of mammalian cells is still the method of choice in biotechnology (O’Flaherty et al., 2020).

Generally, transfection methods can be divided into biological, physical and chemical, and within biological methods we can distinguish viral, nonviral, or a combination of both methods (Chong et al., 2021). Virus-based transfection, more specifically known as transduction, involves the use of a viral vector to carry a specific nucleic acid sequence into a host cell. Retroviruses, such as lentiviruses, are often used for stable transfection (Fakhiri et al., 2019). In contrast, adenovirus, adeno-associated virus (AAV), and herpes virus are viral vectors that do not guarantee stable transfection (Lee et al., 2017). Viral transduction assures high protein yield, but is often associated with increased cytotoxicity and may pose a risk for viral infection (Chong et al., 2021). On the other hand, nonviral transfection methods can be divided into physical methods (such as magnetoreception, electroporation, delivery with biolistic particles, and laser-mediated transfections) and chemical methods, each with distinct advantages and limitations.

Chemical methods are the most widely used methods, showing low cytotoxicity effects, user-friendliness, and cost-effectiveness, with no mutagenesis, no viral vector involvement, no size limitation on the packaged nucleic acid, and no safety problems (Mancinelli et al., 2021). Chemical methods consist mostly of cationic polymers or cationic lipids which, in the presence of DNA, spontaneously form self-assembled complexes through electrostatic interaction (Sharma et al., 2021). The success of chemical transfection depends on the formation of these complexes, which are attracted to the negatively charged cell membrane passing through it, with mechanisms involving endocytosis and phagocytosis. Intracellularly, endocytosed complexes are subsequently transported to the subcellular membrane structure endosomes and/or lysosomes, with some complexes escaping lysosomal degradation and entering the nucleus (Cardarelli et al., 2016).

Cationic lipids consist of amphiphilic molecules with a positively charged headgroup, a hydrophobic domain and a linker connecting the polar head group with the non-polar tail. Their nature correlates with the biocompatibility (Sharma et al., 2021). Commercially available FuGENE 6^®^, for example, is a non-liposomal 100% synthetic lipid-based multi-component transfection reagent designed to transfect plasmid DNA into a wide variety of cell lines with high efficiency (Cocchiararo et al., 2022). Another widely used cationic lipid is Lipofectamine^®^, a reagent that forms complexes (lipoplexes) with nucleic acids, facilitating their cellular uptake via endocytosis. Lipofectamine® has been successfully applied for the transfection of DNA, RNA, and siRNA in various mammalian cell lines, offering high transfection efficiency and versatility. However, the high costs of FuGene 6®, Lipofectamine^®^ and other widely used transfection reagents are a substantial economic bottleneck (Ojha et al., 2025; Carreño et al., 2024).

Cationic polymers are polycations containing different amino groups which bind to the negatively charged phosphate groups of the nucleic acids forming polyplexes (Cai et al., 2023). The most common systems are linear or branched polyethyleneimine (PEI) polymers (Cai et al., 2023) as the hydrochloride or free-base form, respectively. PEI is widely used due to its low cost, ease of use, and ability to efficiently condense nucleic acids into positively charged complexes that facilitate endocytic uptake. Moreover, it enables high transfection efficiency in many cell types, particularly in suspension cultures and large-scale applications such as protein production. However, PEI can be cytotoxic, especially at high concentrations or with prolonged exposure, and often requires careful optimization of the DNA-to-reagent ratio to minimize cellular stress while maintaining transfection efficiency (Mancinelli et al., 2015; 2021). PEI is commercialized in both linear and branched forms, each available in various molecular weights (Lungu et al., 2016). For example, branched PEI is commonly sold in 25 or 50 kDa formulations (Shen et al., 2020). The commercially available PEI MAX® from Polysciences is based on linear PEI hydrochloride (40 kDa) and is known for its high transfection efficiency combined with low cytotoxicity (Delafosse et al., 2016). Particularly, has been reported the synthesis of linear PEI from poly (2-ethyl-2-oxazoline), resulting in polymers with molecular weights ranging from 10 to 87 kDa in the free-base form (Lázaro-Martínez et al., 2015). Specifically, PEI.HCl polymer, with a molecular weight of 87 kDa as free base, referred as PolyAr87, is synthesized from the corresponding 200 kDa poly (2-ethyl-2-oxazoline) (Lázaro-Martínez et al., 2015). This reagent, which is provided in a ready-to-use form, has been successfully used for the expression of the receptor-binding domain (RBD) of the SARS-CoV-2 virus in the development of equine hyperimmune serum therapy for COVID-19 (Zylberman et al., 2020) and for the development of Anti-RBD In-House ELISA (Tomas-Grau et al., 2021), as well as for the transfection of bovine fetal fibroblasts in Argentina (Forcato et al., 2017). More recently it has also been selected for transfection of human melanoma cells to study the impact of the interferon pathway on different therapeutic responses (Mentucci et al., 2024). Due to its lower cost compared to commercial transfection reagents, including PEI-based ones, PolyAr87 could be an advantageous transfectant for a wide range of assays. Chemical transfection efficiency depend on cell type, genetic material to be introduced, and chemical method adopted, and is largely affected by several parameters, both quantitative and qualitative (Mancinelli et al., 2021). Here, we present the optimization of a PolyAr87-based method for the transfection of HEK293T cells and the production of specific llama-derived single-domain antibodies -commonly referred as nanobody- against noroviruses (Garaicoechea et al., 2015; Salmen et al., 2023). Nanobodies offer several advantages over traditional antibodies for immunotherapeutic applications: they are significantly smaller in size (∼15 kDa), demonstrate enhanced thermal stability, and exhibit increased resistance to proteolytic degradation (Salmen et al., 2023). Nanobodies are considered the smallest particles capable of binding to an antigen so they can reach hidden epitopes that conventional antibodies cannot (Rizk et al., 2024). Nanobodies fused to horseradish peroxidase result in a bifunctional molecule retaining both antigen-binding and enzymatic activity, which is highly relevant for ELISA and other immunoassays (Llauger et al., 2021; Vega et al., 2021; Zhao et al., 2022). Moreover, nanobodies can be expressed in different platforms like bacteria, yeast, or mammalian cells (Wu, 2025); however, the mammalian cells expression system is not well studied for nanobody expression.

For the method optimization, we applied a Design of Experiments (DoE) approach. DoE is extremely suitable for the optimization of multivariable assays; unlike the traditional one-factor-at-a-time (OFAT) method, DoE allows simultaneous modulation of multiple factors, revealing potential interactions between variables and ultimately defining the best factor combination for process optimization (Myers et al., 2016). DoE has been efficiently applied across diverse cell-biology workflows, including transfection and toxicity assays (Mancinelli et al., 2015; Elshereef et al., 2019; González-Domínguez et al., 2022; Liguori and Kisslinger, 2022; Kumara et al., 2023; Rampado and Peer, 2023; Carreño et al., 2024; Tzimou et al., 2024; Guo et al., 2025). Thus, recent advances in this field have further demonstrated the power of DoE-derived models to refine transfection performance in next-generation expression systems. For instance, Guo et al. (2025) applied hybrid factorial-RSM schemes to optimize nanoparticle-mediated delivery in suspension cultures, achieving substantial gains in both uptake and viability. Tzimou et al. (2024) used DoE-guided optimization to benchmark novel polymeric transfectants, showing that DoE can accurately predict nonlinear effects associated with polymer-to-DNA stoichiometry and complexation kinetics. Carreño et al. (2024) expanded DoE applications to high-throughput CRISPR delivery platforms, confirming that DoE improves reproducibility and reduces reagent usage compared to OFAT. Meanwhile, Rampado & Peer (2023) and Liguori and Kisslinger (2022) highlighted that multilevel DoE designs (e.g., mixture and definitive screening designs) are increasingly used to characterize physicochemical–biological interactions in complex transfection systems, enabling fine-tuning of particle morphology, charge distribution, and intracellular trafficking. Collectively, these recent reports confirm that DoE now represents the state-of-the-art framework for predictive, resource-efficient optimization of transfection workflows. More specifically, a transfection-specific DoE framework termed Design of Transfection (DoT) was previously developed to optimize a PEI-based transfection protocol for neural progenitors, demonstrating that linear PEI outperforms branched PEI in this context (Mancinelli et al., 2021). In the present study, after evaluating the performance of PolyAr87 against other commercially available transfection reagents, we applied the DoT workflow to analyze, model, and optimize PolyAr87-mediated transfection, providing the first systematic DoE-based protocol for this reagent and extending DoT to a distinct cell type and expression system involving the expression of nanobodies fused to horseradish peroxidase. We employed a response surface methodology (RSM) using a Box–Behnken design (BBD) to identify the optimal combination of factors that maximizes transfection efficiency and to develop a predictive model describing the relationship between performance and its most influential variables. This model was subsequently validated. Our data demonstrate both the efficacy of PolyAr87 as a transfection reagent and the robustness and transferability of the DoT framework for optimizing cell-transfection assays across different reagents, cell contexts, and expression targets.

## 2 Materials and methods

### 2.1 Cell culture and plasmids

The experiments were conducted using a working cell bank (50 cryovials) of HEK293T cells (Merck), which was obtained after 6 passages (7th generation) from the first thawed cryovial from the master cell bank. Furthermore, thawed cryovials from the working bank were sub-cultured at least 4 times before each transfection experiment started. HEK293T cells were thawed, cultured, and sub-cultured in Dulbecco’s modified Eagle’s medium (DMEM, Gibco) supplemented with 10% inactivated fetal bovine serum and 2 mM L-glutamine (Gibco). Opti-MEM (Gibco) was used for the transfection steps. The plasmid used was a pcDNA3.1-MYC-HIS A backbone producecd by GenScript, carrying the coding sequence for a nanobody directed to the P domain of the VP1 capsid protein of Human Norovirus GI.1 genotypes -nanobody N2 (Garaicoechea et al., 2015)- linked to a coding sequence for horseradish peroxidase (HRP), under the influence of the Cytomegalovirus (CMV) promoter, with a 5′ IL-2 secretion signal sequence: pcDNA3.1_NbN2-HRP (Supplementary Figure S1). The HRP sequence used was slightly modified from erHRP (Yamagata and Sanes, 2018).

### 2.2 PolyAr87 synthesis

PolyAr87, a 87 kDa PEI·HCl polymer in its free-base form, was prepared by dissolving 0.11 g of lyophilized powder—obtained after treating 200 kDa poly (2-ethyl-2-oxazoline) with 24% HCl under reflux—in 100 mL of water. PolyAR87 was aliquoted and stored at −80 °C until use.

### 2.3 Transfection assay

The day before the transfection, the cells were seeded in a 24-well plate at 5 × 10^5^ cells/well in a final volume of 0.5 mL of supplemented DMEM per well. On the day of transfection, with cell confluence between 80% and 100%, experiments were conducted as follows: different amounts of DNA and transfection reagents were mixed in 100 µL of prewarmed Opti-MEM, or dissolved separately in 50 µL of prewarmed Opti-MEM each and then mixed. DNA and transfection reagent mixtures were then vortexed and incubated at room temperature for 5–30 min. Meanwhile, the DMEM was removed from the cell cultures, and each well was filled with 0.5 mL of Opti-MEM. The transfection mixtures were then added dropwise to each well under circular movements. The plates were incubated at 37 °C under 5% CO2 for 4 h, after which the serum-free Opti-MEM was replaced with the original growth medium (DMEM supplemented with 10% heat-inactivated fetal bovine serum and 2 mM L-glutamine). Samples from the supernatants were removed at 24 h post-transfection (hpt) and analyzed as follows: 15 µL of each supernatant was mixed with 40 µL of 3.3′,5.5′,tetramethylbenzidine (TMB) at room temperature; after 50 s, the reaction was stopped by adding 50 µL of 1N H2SO4. After 5 min, absorbance at 450 nm (A450) was measured as an indicator of the amount of HRP-conjugated nanobody and then of the transfection efficiency obtained. The colorimetric reaction, in fact, is directly proportional to the amount of expressed HRP-associated nanobody. The outcomes, main factor effects, and any possible interactions were analyzed via Minitab software.

### 2.4 Comparison of PolyAr87 with different transfection reagents

We compared PolyAr87 with other commercially available polycationic reagents: 40 kDa linear PEI Max (Polyscience Inc.) and 25 kDa branched PEI (Sigma-Aldrich) –both powder forms for preparation of the transfection reagents–and FuGene 6® (Promega), for transfection of HEK293T cells. pcDNA3.1_NbN2-HRP plasmid was used for all the transfection experiments. The assays were conducted using the same batch of HEK293T cells in 6-well plates. Briefly, branched PEI Sigma, PEI-Max, PolyAr87 and FuGene 6® were tested at 1 µg/mL plasmid and 3 µg/mL reagent, using one plate each one, per duplicate. The transfection efficiencies for transfection assays were measured as described above, by analytical triplicate. Additionally, the Expi293F suspension cell line was used to compare the transfection efficiencies of PEI Max and PolyAr87. Cells were cultured in 25 mL of Expi293™ Expression Medium in 125-mL shake flasks and subcultured at 1.0 × 10^7^ cells/mL for four passages prior to the transfection experiments. PolyAr87 and PEI Max were tested at 3 µg/mL in the presence of 1 µg/mL of pcDNA3.1_NbN2-HRP plasmid when the cultures reached 3.0 × 10^6^ cells/mL, in duplicate. Transfection efficiencies were quantified as described above, in analytical triplicate.

### 2.5 DoE

To find the best factor combinations via PolyAr87, a design of experiment (DoE) method was applied. Following the DoT workflow conducted by Mancinelli et al. (2021), a full factorial design (FFD) followed by a Box–Behnken design (BBD) was performed. For factors and level selection, exhaustive bibliographic research was performed (see Results and Discussion). The design of the experiments and the data analyses were performed via Minitab Statistical Software version 18 (www.minitab.com; Minitab Inc.).

#### 2.5.1 Full factorial design

**FIGURE 1:**
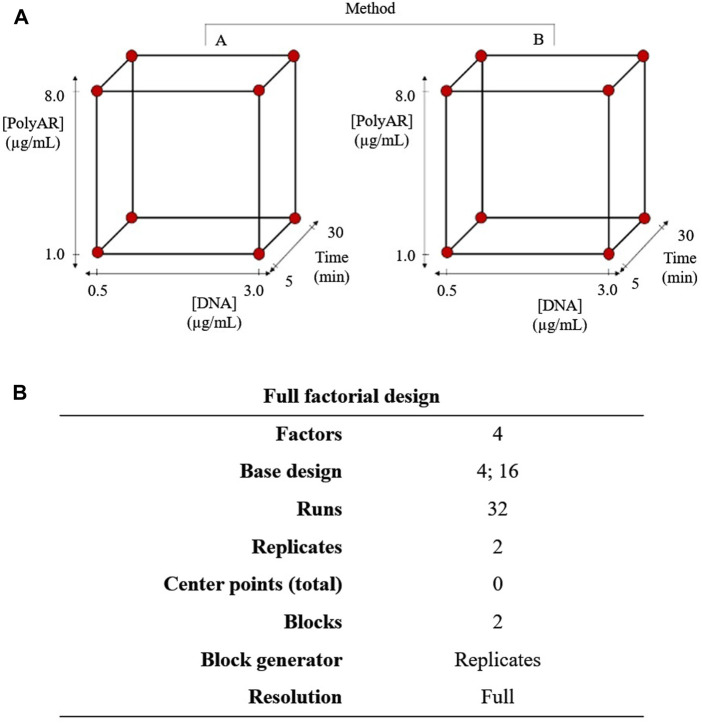
**(A)** Scheme of experimental design for FFD. **(B)** Features of the experimental design for the FFD.

A Full Factorial Design (FFD) allows users to analyze the effect on the response output of each factor as well as the interactions between them (Myers et al., 2016). The experimental design chosen was a two-level FFD, including 2k different combinations (where k is the number of factors analyzed) and two values or levels, coded as high (+1) and low (−1) for each factor (Mancinelli et al., 2021). The four factors and the corresponding levels chosen were as follows: i. DNA concentration (0.5–3.0 μg/mL); ii. PolyAr87 concentration (1.0–8.0 μg/mL); iii. transfection method (A: dissolving DNA and PolyAr87 separately and then mixing both solutions; B: dissolving DNA and PolyAr87 together in the same solution); and iv. incubation time (5–30 min), as schematized in Figure 1A. Thus, the design studied three quantitative factors and one qualitative factor (transfection method) and had 2^4^ = 16 different combinations. Since the test was performed in duplicate, 32 experiments were performed. Duplicates were identified by separate blocks to account for any differences in experimental conditions, running blocks on different days in 24-well plates. Features of the FFD are shown in Figure 1B. 24 hpt was the time selected to measure the transfection output. Minitab software was used to generate the design worksheet shown in Supplementary Table S1.

#### 2.5.2 Box–Behnken design

**FIGURE 2:**
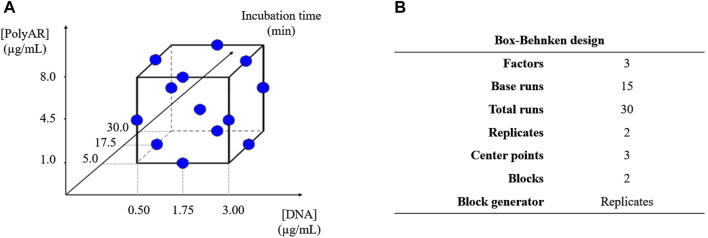
**(A)** Scheme of experimental design for BBD. **(B)** Features of the experimental design for the BBD.

The *Box–Behnken* Design (BBD) included a number of combinations *N = 2k (k – 1) + C0*, where *k* is the number of factors, and *C0* is the number of central points (de Albuquerque Mendes et al., 2024). Three factors were chosen according to the results of the previous experiments and tested: i. the concentration of the PolyAr87 transfection reagent (1.0–8.0 μg/mL), ii. the concentration of plasmid DNA (0.5–3.0 μg/mL), and iii. the complex formation time (5–30 min), as schematized on Figure 2A. As prescribed by the method, three central points were added. Therefore, according to the formula, the number of tests to perform was 15. Since each test was performed in duplicate, a total of 30 experiments were performed. Duplicates were identified in separate blocks to account for any differences in experimental conditions. Features of the FFD are shown in Figure 2B. Minitab software was used to generate the design worksheet, which is shown in Supplementary Table S2 and provides a statistical analysis of the experimental outcome.

#### 2.5.3 BBD model validation

To validate BBD, nine different combinations were tested in duplicate, following the same procedure as for BBD, in two completely independent blocks, which are shown in Supplementary Table S3; each block was performed on different days, starting from different cell cultures. Transfection efficiencies were quantified at 24 hpt as described above, in analytical triplicate. The resulting absorbance averages were compared with the predicted outcomes from the BBD model. Non-significant factors were kept constant during model validation, using the values previously optimized in our laboratory.

## 3 Results and discussion

### 3.1 Comparison of PolyAr87 with different transfection reagents

**FIGURE 3:**
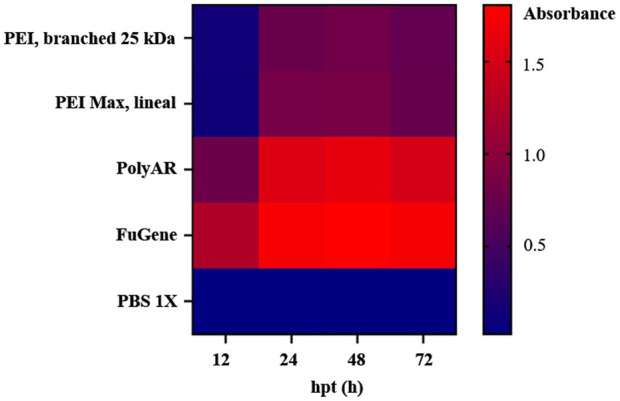
Heatmap of the comparative transfection assays of PolyAr87 with other widely used transfectant reagents (40 Kad PEI MAX, 25 kDa branched PEI and FuGene 6®), using recommended protocols. Activity measurements were conducted at different hours post-transfection (hpt): 12, 24, 48 and 72 hpt. The experiments were conducted using pcDNA3.1_NbN2-HRP plasmid, in one well per each transfectant reagent, by duplicate. After each hpt, the supernatants were separated and mixed with TMB. The absorbances were measured at 450 nm. The absorbance values (averages from analytical triplicates) are expressed on a color scale, with the lower values indicated with blue and the higher values indicated with red.

To evaluate the relative performance of PolyAr87, we first conducted a comparative analysis against commonly used transfection reagents. As is shown in Figure 3, the heatmap obtained from the comparative transfection assays shows that PolyAr87 achieves efficiencies higher or comparable to those of the other commercial transfection reagents tested. To ensure a consistent basis for comparison across all systems, we evaluated each reagent using a fixed DNA amount of 1 µg/mL and a 1:3 DNA-to-reagent ratio. This ratio was selected as a standard reference point within the recommended working range for 40 kDa PEI Max (Longo et al., 2013; Connor Payne and Mazitschek, 2022), 25 kDa branched PEI (Raymond et al., 2011), and FuGene 6® (Promega Corporation, 2025), as it has been found optimal for many genes and cell lines described in the different protocols. Nevertheless, it is well established that the optimal ratio can vary depending on the construct, and recommended practice is to screen a broader window of conditions, typically between 1:1 and 1:5 (Longo et al., 2013; de los Milagros Bassani Molinas et al., 2014; Mancinelli et al., 2021; Connor Payne and Mazitschek, 2022; Kumara et al., 2023; Carreño et al., 2024). Our selection of the 1:3 ratio provided a robust and standardized starting point for comparing PolyAr87 with the other transfection reagents, under equivalent conditions. Additionally, as a clarification, it was beyond the scope of the present study to evaluate all transfection reagents currently available on the market; therefore, we focused on reagents that are broadly accessible to most laboratories, including those outside high-income settings. For this reason, more recently developed reagents such as FectoPRO or PEIpro were not included in our comparative analysis.

In this context, PolyAr87 (having a cost of USD 100/mL) demonstrated expression levels slightly lower than those achieved with others ready-to-use transfectant reagents such as FuGene® 6 (USD 1000/mL), but substantially higher than those obtained with both linear and branched PEIs prepared from powder, when compared under their recommended conditions using a 1:3 DNA-to-reagent ratio.

Consequently, since PolyAr87 proved to be an efficient transfection agent, we next applied a DoE approach to systematically optimize its performance across key transfection parameters.

### 3.2 DoE

DoE is an immensely powerful tool for optimizing cellular assays, including transfection and, consequently, increasing the yield of produced exogenous protein. Particularly, a DoT model has been specifically developed for cell transfection assay and validated to optimize a PEI-based protocol on neural progenitor cells, which are known to be hard to transfect (Mancinelli et al., 2021). Some key points to consider include the identification of the key factors that can affect the process, such as culture conditions, transfection methods, transfectant concentration, and stoichiometric relationships, as well as the identification of the levels for each factor selected. The matrix design facilitates the application of a systematic approach, incorporating full-factorial design and response surface analysis (RSM). Finally, the statistical methods and software available for analyzing the experimental data obtained are powerful for identifying significant factors, interactions, and optimal conditions for protein expression.

#### 3.2.1 Full factorial design

A two-level Full Factorial Design (FFD) was used to identify the parameters that significantly affect transfection and its interactions. The factors to be tested in the FFD and the relative levels are crucial. Here we report the four factors we have chosen, three quantitative and one qualitative, and the related factor levels (low or −1 and high or +1).

##### 3.2.1.1 Factors i and ii: concentration of nucleic acid and concentration of transfection reagent

Concentration of nucleic acid, concentration of transfection reagent and the ratio between both are well known to influence transfection efficiency in mammalian cells (Cocchiararo et al., 2022; González-Domínguez et al., 2022; Carreño et al., 2024; Jamour et al., 2024). Both have been tested as variables in DoT workflow as well as other DoE approaches (Thompson et al., 2012; Elshereef et al., 2019; Mancinelli et al., 2021). The DoT model showed that DNA and PEI concentration are two key factors that influence output in PEI-based transfection of mammalian cells (Mancinelli et al., 2021). Moreover, optimal DNA concentration for transfection of CHO and HEK cells using PEIs ranged from 1.0 μg/mL to 2.5 μg/mL or more, with some of them reporting even 0.5 μg/mL, and with a maximum at 1.5 μg/mL, based on several reported outcomes on literature (Muller et al., 2007; Liu et al., 2008; Ye et al., 2009; Zhang et al., 2009; Bollin et al., 2011; Carreño et al., 2024). The DoT model explored a DNA concentration range between 0.5 and 2.5 μg/mL, finding the better output at lower concentrations (Mancinelli et al., 2021). Then, we decided to choose 0.5 μg/mL as −1 level and 3.0 μg/mL as +1 level. On the other hand, optimal DNA transfection reagent ratio used was reported to be 1:3 in several studies and protocols (Mancinelli et al., 2021; Carreño et al., 2024; de los Milagros Bassani Molinas et al., 2014; Longo et al., 2013; Connor Payne and Mazitschek, 2022; Kumara et al., 2023), with a range varying from 1:1 to 1:6.5. Since the maximum yield of protein has been mostly achieved around 1.5 μg/mL of DNA, we decided to choose 8.0 μg/mL as +1 level for transfection reagent (approximately 5 times more). Based on previous DoE assays on PEI cell toxicity (Mancinelli et al., 2015), we decided not to pass this level to avoid dramatic reduction of protein expression and transfection efficiency due to PEI induced cell death. Finally, to explore a broader interval we choose 1.0 μg/mL as −1 level.

##### 3.2.1.2 Factor iii: method of polymers formation

Variations in polymer incubation protocols reported in the literature prompted us to evaluate two different approaches for PolyAr87 complex formation: (A) preparing separate solutions of DNA and PolyAr87, followed by their mixing, as described in previous studies (Mancinelli et al., 2021; Longo et al., 2013; Fang and Shen, 2010); and (B) dissolving DNA and PolyAr87 together directly in a single solution, as reported in other protocols (L’Abbé et al., 2018; González-Domínguez et al., 2019; Carreño et al., 2024).

##### 3.2.1.3 Factor iv) incubation time

A wide range of DNA–PEI incubation times has been reported in the literature, varying from 10 to 30 min (Carreño et al., 2024; Fang and Shen, 2010; L’Abbé et al., 2018; González-Domínguez et al., 2019; Longo et al., 2013). Therefore, we decided to test this parameter, defining 5 min as the −1 level and 30 min as the +1 level.

**FIGURE 4:**
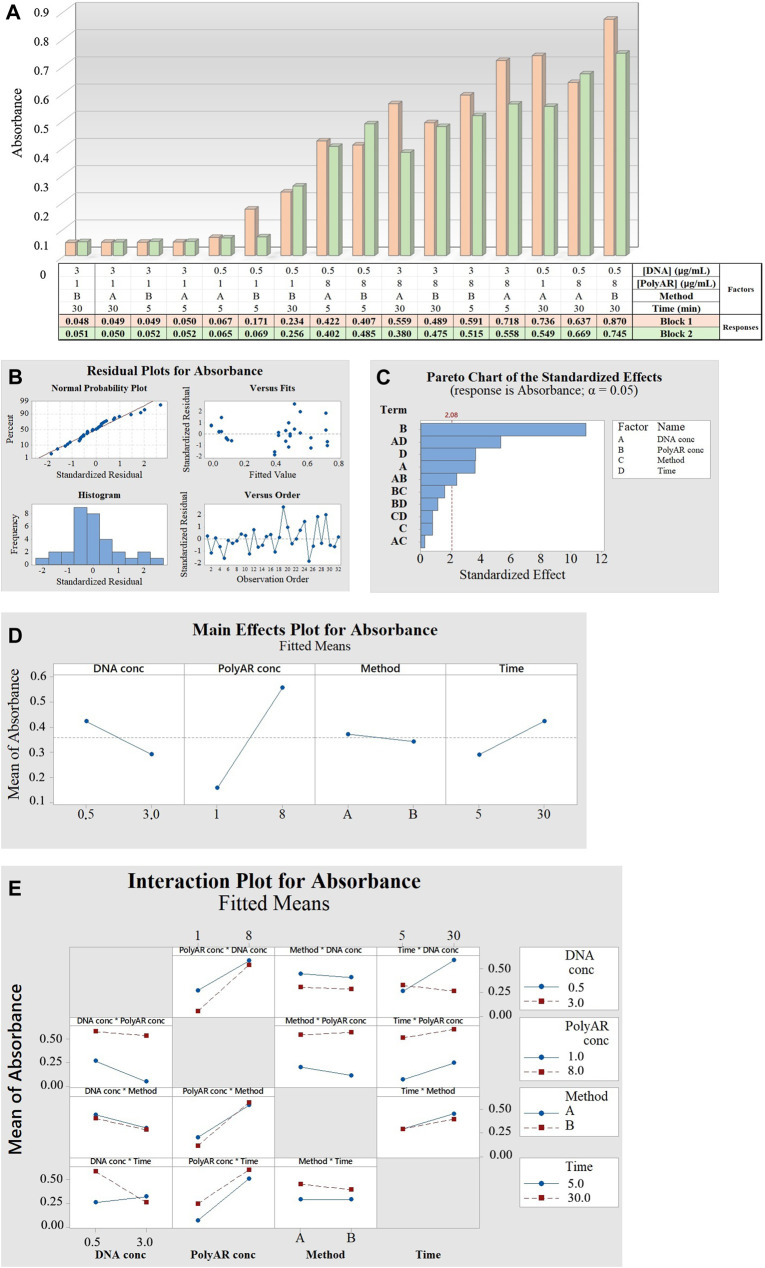
**(A)** Bar plot showing the response outcomes (transfection efficiencies) obtained for the 16 different combinations analyzed in replicates (block 1 and block 2). **(B)** Normal probability plot, standardized residual versus fitted values, histogram and standardized residual versus observation order. **(C)** Pareto chart of the standardized effects. Significance is shown for factors B, D and A, and significance for interactions AD and AB. **(D)** Plot of the main effects on the absorbance. **(E)** Interaction plot for absorbance.

The FFD analysis enabled the determination of the optimal conditions that maximize Transfection efficiency (Mancinelli et al., 2021). The two-level FFD included four factors, one qualitative (transfection method) and three quantitative (DNA concentration, PEI concentration and complexation time) with two levels each (−1; +1). The design included 16 different run combinations, resulting in 32 since the tests were performed in duplicate (in different blocks). The response outputs (A450) measured after 24 hpt (Figure 4A), varied from 0.048 to 0.870, indicating a normal distribution and constant variance of the residuals (Figure 4B). Additionally, as shown in the factorial regression analysis (Table 1) and in the Pareto chart (Figure 4C), 3 out of the 4 tested factors had a significant effect on transfection (DNA concentration, PolyAr87 concentration, and complexation time), with the PolyAr87 concentration being the most influential factor (effect = 0.3984; *p* < 0.0001), followed by complexation time (effect = 0.1327; *p* < 0.005) and DNA concentration (effect = −0.1311; *p* < 0.005). Interestingly, the output increases at higher concentration of transfectant and lower concentration of DNA (Figure 4D), suggesting that higher PEI/DNA ratios are favored in these intervals, in line with previous studies (Mancinelli et al., 2021). In this sense, from Figure 4A, 7 out of 8 conditions showing the higher outcomes were run with the +1 level (8.0 μg/mL) of PolyAr87. Additionally, the effect of complexation time was positive, indicating that longer DNA–PolyAr87 interaction periods favor complex stabilization and improved expression. In contrast, the transfection method exerted only a minimal influence on the outcome, consistent with the lack of statistical significance observed in the regression model. Furthermore, if we analyze the two-way interactions for fitted means (Figure 4E), only two interactions were significant: DNA concentration × complexation time (effect = −0.1932; *p* < 0.0001) and DNA concentration × PolyAr87 concentration (effect = 0.0871; *p <* 0.05). The negative interaction between DNA concentration and complexation time suggests that longer complexation times may be detrimental when higher DNA levels are used, potentially due to the formation of overly compacted or unstable complexes that hinder cellular uptake. In contrast, the positive interaction between DNA and PolyAr87 concentrations indicates that the adverse effect of increasing DNA levels can be partially mitigated by using higher amounts of transfectant. This supports the idea that the PEI/DNA ratio plays a critical role, and that adjusting one factor without considering the other may lead to suboptimal results. These findings underscore the importance of balancing these variables together rather than optimizing them in isolation.

**TABLE 1:** Factorial regression for the FFD.

Term	Effect	Coeff	T value	P value
Constant	​	0.3584	20.02	0.0000
Blocks	​	0.0226	1.26	0.2208
DNA conc	−0.1311	−0.0656	−3.66	**0.0015**
PolyAr87 conc	0.3984	0.1992	11.13	**0.0000**
Method	−0.0285	−0.0142	−0.80	0.4353
Complexation time	0.1327	0.0664	3.71	**0.0014**
DNA conc*PolyAr87 conc	0.0871	0.0436	2.43	**0.0245**
DNA conc*Method	0.0102	0.0051	0.29	0.7776
DNA conc*Time	−0.1932	−0.0966	−5.40	**0.0000**
PolyAr87 conc*Method	0.0575	0.0287	1.61	0.1239
PolyAr87 conc*Time	−0.0420	−0.0210	−1.17	0.2545
Method*Time	−0.0291	−0.0146	−0.81	0.4255

P values lower than 0.05 were considered statistically significant and are highlighted in bold.

Finally, the factor iii) transfection method did not have a significant effect on transfection (effect = −0.0285; *p* < 0.4353). Blocks, set to investigate possible influences of non-modelled factors on the response, did not show significance (*p* = 0.2208), proving that the experiments were performed in equivalent conditions. Therefore, the factors PolyAr87 concentration, DNA concentration, and incubation time, whose effects were established as significant regarding the efficiency of nanobody expression, were further investigated in DoT optimization. In this new experiment, the transfection method was kept constant, and the subsequent experiments were all performed via method B (dissolving both nucleic acid and transfectant directly in the same mixture) for simplicity.

#### 3.2.2 Box-Behnken design

**FIGURE 5:**
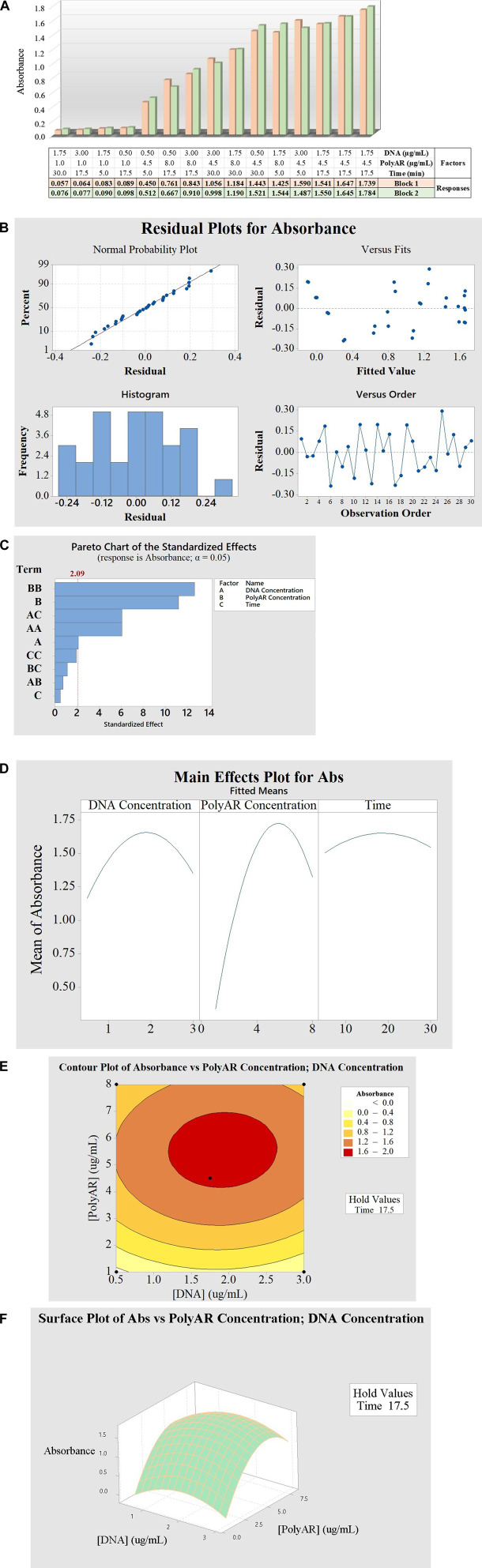
**(A)** Bar plot showing the response outcomes (transfection efficiencies) obtained for the different analyzed combinations. **(B)** Normal probability plot, standardized residual values versus fitted values, histogram, and standardized residual values versus observation order. **(C)** Pareto chart of the standardized effects, showing significance for factors B and A, and significance for interactions BB, AC and AA. **(D)** Plot of the main effects on the absorbance. **(E)** 2D contour plot of absorbance vs. PolyAr87 concentration and DNA concentration. **(F)** 3D surface plot of absorbance vs. PolyAr87 concentration and DNA concentration.

A BBD was used for further optimization, as suggested by the DoT model (Mancinelli et al., 2021). In this case, three factors were tested: i. the concentration of PolyAr87, ii. the concentration of plasmid DNA, and iii. the complex formation time. The response outputs (A450), which are shown in Figure 5A, varied from 0.057 to 1.784, indicating a normal distribution and constant variance of the residuals (Figure 5B). Additionally, as shown in the analysis of variance (Table 2), in the response surface regression for BBD (Table 3), in the Pareto chart (Figure 5C), and in the effect plots for absorbance (Figure 5D), 2 out of 3 of the factors tested had a significant effect on transfection (PolyAr87 concentration and DNA concentration), with the PolyAr87 concentration being the most influential factor (adj MS = 3.8908; *p* < 0.0001), followed by the DNA concentration (adj MS = 0.1376; *p* < 0.05). Furthermore, 3 interactions significantly affected the transfection efficiency: PolyAr87 concentration × PolyAr87 concentration (adj MS = 4.9556; *p* < 0.0001), DNA concentration × complexation time (adj MS = 1.1438; *p* < 0.0001), and DNA concentration × DNA concentration (adj MS = 1.1416; *p* < 0.0001). Interestingly, the factor complex formation time did not have a significant effect (adj MS = 0.0074; *p* > 0.05) as a main factor, while it is significant in interaction with DNA concentration.

**TABLE 2:** Analysis of variance for BBD.

Source	DF	Adj SS	Adj MS	F-Value	P-Value
Model	10	11.0132	1.1013	35.63	**0.0000**
Blocks	1	0.0010	0.0010	0.03	0.8561
Linear	3	4.0358	1.3453	43.52	**0.0000**
DNA Concentration	1	0.1376	0.1376	4.45	**0.0483**
PolyAR Concentration	1	3.8908	3.8908	125.87	**0.0000**
Time	1	0.0074	0.0074	0.24	0.6303
Square	3	5.7768	1.9256	62.30	**0.0000**
DNA Concentration*DNA Concentration	1	1.1416	1.1416	36.93	**0.0000**
PolyAR Concentration*PolyAR Concentration	1	4.9556	4.9556	160.32	**0.0000**
Time*Time	1	0.1167	0.1167	3.77	0.0670
2-Way Interaction	3	1.1995	0.3998	12.94	**0.0001**
DNA Concentration*PolyAR Concentration	1	0.0172	0.0172	0.56	0.4648
DNA Concentration*Time	1	1.1438	1.1438	37.00	**0.0000**
PolyAR Concentration*Time	1	0.0385	0.0385	1.25	0.2783
Error	19	0.5873	0.0309		
Lack-of-Fit	15	0.5400	0.0360	3.04	0.1457
Pure Error	4	0.0473	0.0118		
Total	29	11.6005			

P values lower than 0.05 were considered statistically significant and are highlighted in bold.

**TABLE 3:** Response surface regression for BBD.

Term	Coef	SE Coef	T-Value	P-Value	VIF
**Constant**	1.6510	0.0718	23.00	0.0000	
**Blocks**					
** 1**	-0.0059	0.0321	-0.18	0.8561	1.00
**DNA Concentration**	0.0928	0.0440	2.11	0.0483	1.00
**PolyAR Concentration**	0.4931	0.0440	11.22	0.0000	1.00
**Time**	0.0215	0.0440	0.49	0.6303	1.00
DNA Concentration*DNA Concentration	-0.3932	0.0647	-6.08	0.0000	1.01
PolyAR Concentration*PolyAR Concentration	-0.8192	0.0647	-12.66	0.0000	1.01
Time*Time	-0.1257	0.0647	-1.94	0.0670	1.01
DNA Concentration*PolyAR Concentration	0.0464	0.0622	0.75	0.4648	1.00
DNA Concentration*Time	-0.3781	0.0622	-6.08	0.0000	1.00
PolyAR Concentration*Time	-0.0694	0.0622	-1.12	0.2783	1.00

P values lower than 0.05 were considered statistically significant and are highlighted in bold.

To better visualize those effects, both 2D and 3D contour plots of the absorbance vs. the PolyAr87 concentration and the DNA concentration are shown in Figures 5E,F, respectively. Notably, the maximum efficiency obtained in the BBD experiments was higher than that one obtained by FFD, indicating that the transfection process has been optimized. Particularly, we observed the highest levels of absorbance and inferred the highest level of nanobody expression and transfection efficiency in the range between 1.2 and 2.6 μg/mL of DNA and between 4.0 and 7.0 μg/mL of PolyAr87.

For completeness, it should be clarified that the BBD was selected based on prior experience with the DoT methodology (Mancinelli et al., 2021). Alternative designs, such as a Face-Centered Composite Design (FCCD) or a D-optimal design, were not pursued. An FCCD would have restricted the exploration to the factor space defined by three levels per factor, limiting orthogonality and experimental flexibility. A D-optimal design is typically applied when classical designs are unsuitable due to high factor numbers or non-standard interactions, which were not present in this study. The ability to locate and center the optimal region allowed the completion of the DoE/DoT at this stage, without the need to proceed to further optimization, using, for example, a Central Composite Design, as was necessary in prior DoE studies (Mancinelli et al., 2021).

Thus, the BBD provided an efficient and practical approach to optimize PolyAr87 transfection while demonstrating the broader applicability of DoT to novel expression systems, including nanobodies.

### 3.3 Model validation

**FIGURE 6:**
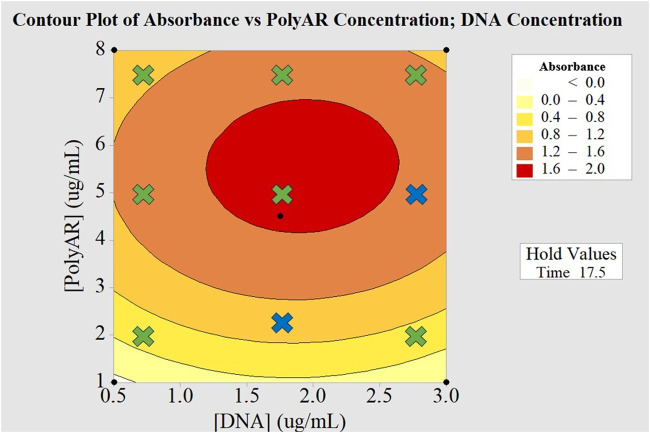
Model validation. The nine tested combinations of PolyAr87 and DNA concentrations are indicated by crosses on the 2D contour plot from Figure 7D. Experiments with outcomes falling within the predicted range are highlighted in green, while those with outcomes slightly exceeding the predicted range are shown in blue.

To validate the predictive accuracy of the BBD, a total of nine experiments were conducted in duplicate using different combinations of DNA and PolyAr87 concentrations, which are shown in Figure 6. Also, the combination values, their respective outcomes, the averages, the standard deviations, and the expected ranges, are shown in Table 4. The experimental results showed a high degree of concordance with the expected outcome ranges provided by the BBD model, indicating that the model successfully captured the relationship between the two variables and the transfection efficiency. Additionally, most of the observed outcome averages aligned with their predicted ranges. Moreover, the standard deviations across duplicates were generally low, ranging from 0.012 to 0.172, indicating good reproducibility across blocks. Particularly, experiments 1, 2, 5, and 6 all demonstrated strong alignment with the expected values, suggesting good model predictability in both low and high concentration regions. Experiment 5 (1.75 µg/mL DNA, 5.0 µg/mL PolyAr87) yielded the highest average response (1.664), closely matching the upper end of the expected range (1.6–2.0), further supporting the robustness of the model at optimal conditions. On the other hand, a few minor deviations were observed: the experiment 4 (1.75 µg/mL DNA, 2.5 µg/mL PolyAr87) yielded a mean of 1.266, just above the expected upper limit (1.2). Also, experiment 8 (2.75 µg/mL DNA, 5.0 µg/mL PolyAr87) yielded a mean of 1.685, just above the expected upper limit (1.6). However, these deviations were small and within two standard deviations of the expected ranges, suggesting that they may result from minor experimental variation rather than a failure in the model itself.

**TABLE 4:** Data from Model validation.

Exp n°	[DNA] (µg/mL)	[PolyAr87] (µg/mL)	Abs outcomes (block 1)	Abs outcomes (block 2)	Outcome averages	Standard deviation	Expected ranges
1	0.75	2.0	0.586	0.624	0.605	0.027	0.4–0.8
2	0.75	5.0	1.121	1.364	1.243	0.172	1.2–1.6
3	0.75	7.5	0.786	0.769	0.778^a^	0.012	0.8–1.2
4	1.75	2.5	1.232	1.299	1.266^b^	0.047	0.8–1.2
5	1.75	5.0	1.620	1.707	1.664	0.062	1.6–2.0
6	1.75	7.5	1.121	1.297	1.209	0.124	1.2–1.6
7	2.75	2.0	0.741	0.875	0.808^b^	0.095	0.4–0.8
8	2.75	5.0	1.637	1.732	1.685^b^	0.067	1.2–1.6
9	2.75	7.5	1.546	1.356	1.451	0.134	1.2–1.6

^a^ Values lower than the expected range.

^b^ Values higher than the expected range.

When comparing our optimal DNA:PEI ratio (maximum expression at 1.75 µg/mL DNA and 5.0 µg/mL PolyAr87, corresponding to a 1:2.86 DNA:PEI weight ratio) with representative studies in HEK-derived systems, Delafosse et al. provide a useful benchmark. In their systematic side-by-side evaluation of several commercial PEIs, they reported that the optimal DNA:PEI weight ratio for HEK293 suspension cells typically centers around 1:3 for both linear PEI and PEI Max, whereas deviations above or below this range lead to reduced productivity and/or increased cytotoxicity (Delafosse et al., 2016). Standard protocols also report similar mass-ratio windows: a PEI:DNA mass ratio between 2:1 and 3:1 is frequently used as an empirical starting point for HEK293/HEK293T transient transfection and is often found to be close to the optimal condition after fine tuning (Addgene, 2023). Kumara et al., who optimized PEI transfections in 293T cells using small-scale reporter assays, directly tested DNA amounts (0.5–1 µg) and mass ratios (e.g., 1:3 vs. 1:4) and reported the highest transfection efficiencies in 293T at the 1:3 ratio, with low toxicity (Kumara et al., 2023). Furthermore, there is few information about nanobody expression in mammalian cells. Van et al., transfected a nanobody-expressing plasmid to control gene expression into HEK293T cells using Lipofectamine 2000 following the manufacturer´s instructions without any ratio optimization protocol (Van et al., 2021). Similarly, Jager et al. expressed scFv-Fc and scFv-Fc-RNase antibodies in HEK293T with high yields using a PEI: DNA ratio of 2.5: 1 (Jäger et al., 2013). Taken together, these references indicate a consistent picture: i. the optimal DNA:PEI mass ratio for HEK derived cells typically lies in the 1:2–1:3 range (w/w), ii. small deviations beyond this window rapidly reduce yield or increase toxicity and iii. the DNA:PEI mass ratio and the absolute amounts of DNA and PEI both impact transfection efficiency. Our optimized PolyAr87 condition (≈1:2.86 w/w) falls squarely within the empirically and mechanistically supported window reported in the literature, while the DoT/response-surface workflow allowed us to refine the precise mass and time parameters specific to PolyAr87 and to our reporter system. On the other hand, the absence of statistically significant differences between the polyplex-formation methods (FFD) and incubation times (BBD) tested in our study is fully consistent with the broad variability and wide operational ranges reported in recent literature (Mancinelli et al., 2021; Connor Payne and Mazitschek, 2022; Carreño et al., 2024; González-Domínguez et al., 2019).

Finally, to contextualize the validation of the optimized PolyAr87 protocol, we compared its performance (1.75 µg/mL DNA, 5.0 µg/mL PolyAr87; Abs = 1.650) with the transfection outcomes of the benchmark reagents at the standard 1:3 DNA:reagent ratio (PEI Max: Abs = 1.089; branched PEI: Abs = 0.785; FuGene 6: Abs = 1.535), as well as with PolyAr87 itself applied at the same 1:3 ratio (Abs = 1.181). This comparison demonstrates the improvement achieved through DoT-based optimization, highlighting that the performance achieved with the optimized PolyAr87 protocol is comparable to the expression levels obtained under the manufacturer-recommended conditions for FuGene 6, even though the latter was not specifically optimized for the current experimental system.

### 3.4 Limitations and scope

Although this study provides a systematic optimization of PolyAr87-mediated transfection, some limitations must be acknowledged. First, all experiments were performed in adherent HEK293T cells cultured in FBS-supplemented medium, which represents the recommended growth condition for this cell line but also a factor known to influence transfection efficiency. Serum deprivation is not ideal for adherent HEK293T cultures, as it can impair attachment, reduce proliferation and viability, and increase cell stress, thereby confounding the interpretation of transfection performance. For this reason, serum concentration was intentionally kept constant in our design and was not explored as an optimization variable. Consequently, the present work should be viewed as a controlled proof of-concept system, and further optimization will be required when adapting PolyAr87 to alternative hosts or to biomanufacturing-relevant environments, such as chemically defined and serum-free suspension cultures. Importantly, to assess the potential applicability of PolyAr87 in industrially relevant settings, we also tested transfection in suspension-adapted Expi293F cells under serum-free conditions. Moreover, Expi293F cells were included as a complementary model to HEK293T cells to evaluate performance in a scalable, industry-relevant expression system widely used for biotherapeutic production. Assessing transfection in a high-density suspension platform allowed us to determine whether the optimized PolyAr87 protocol remains effective under conditions that more closely reflect current trends in industrial protein manufacturing. Thus, transfection efficiency for each reagent was assessed at different time points post-transfection by measuring the absorbance of the Expli293F culture supernatants, corresponding to the enzymatic activity of the expressed HRP using a TMB based chromogenic assay, as shown in Supplementary Figure S2. PolyAr87 achieved transfection efficiencies comparable to commercially available PEI MAX®, demonstrating its versatility and efficiency when using chemically defined, serum-free, suspension culture systems commonly used in biomanufacturing workflows. Finally, although our main objective was to benchmark PolyAr87 against widely used transfection reagents, industrially oriented reagents such as FectoPRO and PEIpro are increasingly implemented in bioproduction workflows (Banos-Mateos et al., 2024; Fu Y. et al., 2024; Fu Q. et al., 2024). Our study did not include direct benchmarking against these reagents, and the available literature is heterogeneous, often based on distinct applications and non-comparable readouts. Therefore, extracting reliable quantitative comparisons is not straightforward. Including these reagents in future work will help further contextualize the performance of PolyAr87 within modern transient expression platforms.

## 4 Conclusion

In recent years, the need for standardized and systematically optimized workflows has become increasingly evident (Bongiovanni et al., 2015; Digilio et al., 2016; Hollmann et al., 2022; Reiter et al., 2021), particularly in transient gene expression, where process robustness and reproducibility remain major challenges (Fu Q. et al., 2024; Tan et al., 2021). Key transfection parameters such as DNA dose, polymer/DNA ratio, and complexation kinetics show strong context dependency, underscoring the value of Design of Experiments (DoE) strategies for resolving complex factor interactions (González-Domínguez et al., 2022; Kumara et al., 2023; Carreño et al., 2024; Mancinelli et al., 2021). However, systematic optimization has been largely overlooked for low-cost PEI-based reagents such as PolyAr87.

Our study fills this gap by demonstrating that PolyAr87 is an effective, economical reagent for transient transfection in both HEK293 and Expi293F cells for nanobody-HRP expression. Using a two-phase DoE/DoT framework, we identified the main determinants of PolyAr87 performance and defined optimized operational conditions. In agreement with previous work on commercial PEIs (Mancinelli et al., 2021; Carreño et al., 2024; de los Milagros Bassani Molinas et al., 2014; Longo et al., 2013; Connor Payne and Mazitschek, 2022; Kumara et al., 2023), DNA and polymer concentrations emerged as the primary drivers of efficiency.

The optimized protocol markedly increased nanobody-HRP expression relative to previously reported standard conditions (Mancinelli et al., 2015; 2021), and model validation confirmed the robustness and predictive accuracy of the optimization strategy. Overall, these findings establish PolyAr87 as a ready-to-use, cost-effective transfection reagent and reinforce DoE/DoT methodologies as powerful and generalizable tools for the rational optimization of transient gene expression workflows in mammalian systems.

Finally, to our knowledge, this is the first study to validate an HRP-fused nanobody expression protocol in HEK293T cells using PolyAr87.

## Data Availability

The original contributions presented in the study are included in the article/Supplementary Material, further inquiries can be directed to the corresponding authors.

## References

[B1] Addgene (2023). General transfection. Available online at: https://www.addgene.org/protocols/transfection/ (Accessed December 10, 2025).

[B64] Bongiovanni A. Colotti G. Liguori G. L. Di Carlo M. Digilio F. A. Lacerra G. (2015). Applying Quality and Project Management methodologies in biomedical research laboratories: a public research network’s case study. Accred. Qual. Assur. 20, 203–213. 10.1007/s00769-015-1132-5

[B2] Banos-Mateos S. Lopez-Robles C. Yubero M. E. Jurado A. Arbelaiz-Sarasola A. Lamsfus-Calle A. (2024). Abolishing retro-transduction of producer cells in lentiviral vector manufacturing. Viruses 16, 1216. 10.3390/v16081216 39205190 PMC11359676

[B3] Bollin F. Dechavanne V. Chevalet L. (2011). Design of experiment in CHO and HEK transient transfection condition optimization. Protein Expr. Purif. 78, 61–68. 10.1016/j.pep.2011.02.008 21354312

[B4] Cai X. Dou R. Guo C. Tang J. Li X. Chen J. (2023). Cationic polymers as transfection reagents for nucleic acid delivery. Pharmaceutics 15, 1502. 10.3390/pharmaceutics15051502 37242744 PMC10223806

[B5] Cardarelli F. Digiacomo L. Marchini C. Amici A. Salomone F. Fiume G. (2016). The intracellular trafficking mechanism of Lipofectamine-based transfection reagents and its implication for gene delivery. Sci. Rep. 6, 25879. 10.1038/srep25879 27165510 PMC4863168

[B6] Carreño A. Guerrero-Yagüe R. Casal E. Mendoza R. Corchero J. L. (2024). Tuning plasmid DNA amounts for cost-effective transfections of mammalian cells: when less is more. Appl. Microbiol. Biotechnol. 108, 98. 10.1007/s00253-024-13003-x 38212965 PMC10784393

[B7] Chong Z. X. Yeap S. K. Ho W. Y. (2021). Transfection types, methods and strategies: a technical review. PeerJ 9, e11165. 10.7717/peerj.11165 33976969 PMC8067914

[B8] Cocchiararo I. Cornut M. Soldati H. Bonavoglia A. Castets P. (2022). Back to basics: optimization of DNA and RNA transfer in muscle cells using recent transfection reagents. Exp. Cell Res. 421, 113392. 10.1016/j.yexcr.2022.113392 36273532

[B9] Connor Payne N. Mazitschek R. (2022). Tiny titans: nanobodies as powerful tools for TR‐FRET assay development. Analysis and Sens. 2, e202200020. 10.1002/anse.202200020

[B10] de Albuquerque Mendes M. K. dos Santos Oliveira C. B. da Silva Medeiros C. M. Dantas C. Carrilho E. de Araujo Nogueira A. R. (2024). Application of experimental design as a statistical approach to recover bioactive peptides from different food sources. Food Sci. Biotechnol. 33, 1559–1583. 10.1007/s10068-024-01540-0 38623435 PMC11016049

[B11] de los Milagros Bassani Molinas M. Beer C. Hesse F. Wirth M. Wagner R. (2014). Optimizing the transient transfection process of HEK-293 suspension cells for protein production by nucleotide ratio monitoring. Cytotechnology 66, 493–514. 10.1007/s10616-013-9601-3 23775287 PMC3973801

[B65] Digilio F. A. Lanati A. Bongiovanni A. Mascia A. Di Carlo M. Barra A. (2016). Quality-based model for Life Sciences research guidelines. Accred. Qual. Assur. 21, 221–230. 10.1007/s00769-016-1205-0

[B12] Delafosse L. Xu P. Durocher Y. (2016). Comparative study of polyethylenimines for transient gene expression in mammalian HEK293 and CHO cells. J. Biotechnol. 227, 103–111. 10.1016/j.jbiotec.2016.04.028 27085888

[B13] Elshereef A. A. Jochums A. Lavrentieva A. Stuckenberg L. Scheper T. Solle D. (2019). High cell density transient transfection of CHO cells for TGF‐β1 expression. Eng. Life Sci. 19, 730–740. 10.1002/elsc.201800174 32624966 PMC6999575

[B14] Fakhiri J. Schneider M. A. Puschhof J. Stanifer M. Schildgen V. Holderbach S. (2019). Novel chimeric gene therapy vectors based on adeno-associated virus and four different mammalian bocaviruses. Mol. Ther. Methods Clin. Dev. 12, 202–222. 10.1016/j.omtm.2019.01.003 30766894 PMC6360332

[B15] Fang Q. Shen B. (2010). Optimization of polyethylenimine-mediated transient transfection using response surface methodology design. Electron. J. Biotechnol. 13, 10–11. 10.2225/vol13-issue5-fulltext-6

[B16] Forcato D. O. Fili A. E. Alustiza F. E. Lázaro Martínez J. M. Bongiovanni Abel S. Olmos Nicotra M. F. (2017). Transfection of bovine fetal fibroblast with polyethylenimine (PEI) nanoparticles: effect of particle size and presence of fetal bovine serum on transgene delivery and cytotoxicity. Cytotechnology 69, 655–665. 10.1007/s10616-017-0075-6 28321779 PMC5507845

[B17] Fu Q. Wang Y. Qin J. Xie D. McNally D. Yoon S. (2024). Enhanced ER protein processing gene expression increases rAAV yield and full capsid ratio in HEK293 cells. Appl. Microbiol. Biotechnol. 108, 459. 10.1007/s00253-024-13281-5 39230729 PMC11374875

[B18] Fu Y. Han Z. Cheng W. Niu S. Wang T. Wang X. (2024). Improvement strategies for transient gene expression in mammalian cells. Appl. Microbiol. Biotechnol. 108, 480. 10.1007/s00253-024-13315-y 39365308 PMC11452495

[B19] Fus-Kujawa A. Prus P. Bajdak-Rusinek K. Teper P. Gawron K. Kowalczuk A. (2021). An overview of methods and tools for transfection of eukaryotic cells *in vitro* . Front. Bioeng. Biotechnol. 9, 701031. 10.3389/fbioe.2021.701031 34354988 PMC8330802

[B20] Garaicoechea L. Aguilar A. Parra G. I. Bok M. Sosnovtsev S. V. Canziani G. (2015). Llama nanoantibodies with therapeutic potential against human norovirus diarrhea. PLoS One 10, e0133665. 10.1371/journal.pone.0133665 26267898 PMC4534396

[B21] Gomes A. R. Byregowda S. M. Veeregowda B. M. Balamurugan V. (2016). An overview of heterologous expression host systems for the production of recombinant proteins. Adv. Anim. Vet. Sci. 4, 346–356. 10.14737/journal.aavs/2016/4.7.346.356

[B22] González-Domínguez I. Grimaldi N. Cervera L. Ventosa N. Gòdia F. (2019). Impact of physicochemical properties of DNA/PEI complexes on transient transfection of mammalian cells. N. Biotechnol. 49, 88–97. 10.1016/j.nbt.2018.09.005 30291892

[B23] González-Domínguez I. Puente-Massaguer E. Lavado-García J. Cervera L. Gòdia F. (2022). Micrometric DNA/PEI polyplexes correlate with higher transient gene expression yields in HEK 293 cells. N. Biotechnol. 68, 87–96. 10.1016/j.nbt.2022.02.002 35151904

[B24] Guo T. J. F. Liang W. Y. Singhera G. K. Memar Vaghri J. Leung J. M. Dorscheid D. R. (2025). Optimization of chemical transfection in airway epithelial cell lines. BMC Biotechnol. 25, 10. 10.1186/s12896-025-00945-x 39849458 PMC11761256

[B66] Hollmann S. Regierer B. D’Elia D. Kisslinger A. Liguori G. L. (2022). Toward the definition of common strategies for improving reproducibility, standardization, management, and overall impact of academic research. Adv. Biomembr. Lipid Self-Assemb. 1–24. 10.1016/bs.abl.2022.05.001

[B25] Jäger V. Büssow K. Wagner A. Weber S. Hust M. Frenzel A. (2013). High level transient production of recombinant antibodies and antibody fusion proteins in HEK293 cells. BMC Biotechnol. 13, 52. 10.1186/1472-6750-13-52 23802841 PMC3699382

[B26] Jamour P. Jamali A. Langeroudi A. G. Sharafabad B. E. Abdoli A. (2024). Comparing chemical transfection, electroporation, and lentiviral vector transduction to achieve optimal transfection conditions in the vero cell line. BMC Mol. Cell Biol. 25, 15. 10.1186/s12860-024-00511-x 38741034 PMC11089686

[B27] Kumara D. Harsan H. S. Novianti M. Lestari D. Septisetyani E. P. Prasetyaningrum P. W. (2023). Optimized condition for pei-based transient transfection of lifeact-gfp/nls-mcherry expressing plasmid used as cell barcode for syncytia live cell imaging. J. Teknosains 13, 99–108. 10.22146/teknosains.89479

[B28] Lázaro-Martínez J. M. Rodríguez-Castellón E. Vega D. Monti G. A. Chattah A. K. (2015). Solid-state studies of the crystalline/amorphous character in linear *poly* (ethylenimine hydrochloride) (PEI·HCl) polymers and their copper complexes. Macromolecules 48, 1115–1125. 10.1021/ma5023082

[B29] Lee C. S. Bishop E. S. Zhang R. Yu X. Farina E. M. Yan S. (2017). Adenovirus-mediated gene delivery: potential applications for gene and cell-based therapies in the new era of personalized medicine. Genes Dis. 4, 43–63. 10.1016/j.gendis.2017.04.001 28944281 PMC5609467

[B30] Liguori G. L. Kisslinger A. (2022). Quality management tools on the stage: old but new allies for rigor and standardization of extracellular vesicle studies. Front. Bioeng. Biotechnol. 10, 826252. 10.3389/fbioe.2022.826252 35360394 PMC8960150

[B31] Liu C. Dalby B. Chen W. Kilzer J. M. Chiou H. C. (2008). Transient transfection factors for high-level recombinant protein production in suspension cultured mammalian cells. Mol. Biotechnol. 39, 141–153. 10.1007/s12033-008-9051-x 18327552

[B32] Llauger G. Monti D. Adúriz M. Romão E. Dumón A. D. Mattio M. F. (2021). Development of Nanobodies against Mal de Río Cuarto virus major viroplasm protein P9-1 for diagnostic sandwich ELISA and immunodetection. Sci. Rep. 11, 20013. 10.1038/s41598-021-99275-z 34625580 PMC8501053

[B33] Longo P. A. Kavran J. M. Kim M.-S. Leahy D. J. (2013). “Transient mammalian cell transfection with polyethylenimine (PEI),” in Methods in enzymology (Elsevier), 227–240. 10.1016/B978-0-12-418687-3.00018-5 PMC4012321 24011049

[B34] Lungu C. Diudea M. Putz M. Grudziński I. (2016). Linear and branched PEIs (polyethylenimines) and their property space. Int. J. Mol. Sci. 17, 555. 10.3390/ijms17040555 27089324 PMC4849011

[B35] L’Abbé D. Bisson L. Gervais C. Grazzini E. Durocher Y. (2018). Transient gene expression in suspension HEK293-EBNA1 cells. Methods Mol. Biol. 1850, 1–16. 10.1007/978-1-4939-8730-6_1 30242676

[B36] Mancinelli S. Zazzu V. Turcato A. Lacerra G. Digilio F. A. Mascia A. (2015). Applying design of experiments methodology to PEI toxicity assay on neural progenitor cells. Math. Models Biol. Bringing Math. Life, 45–63. 10.1007/978-3-319-23497-7_4

[B37] Mancinelli S. Turcato A. Kisslinger A. Bongiovanni A. Zazzu V. Lanati A. (2021). Design of transfections: implementation of design of experiments for cell transfection fine tuning. Biotechnol. Bioeng. 118, 4488–4502. 10.1002/bit.27918 34406655 PMC9291525

[B38] Mentucci F. M. Romero Nuñez E. A. Ercole A. Silvetti V. Dal Col J. Lamberti M. J. (2024). Impact of genomic mutation on melanoma immune microenvironment and IFN-1 pathway-driven therapeutic responses. Cancers (Basel) 16, 2568. 10.3390/cancers16142568 39061208 PMC11274745

[B39] Muller N. Derouazi M. Van Tilborgh F. Wulhfard S. Hacker D. L. Jordan M. (2007). Scalable transient gene expression in Chinese hamster ovary cells in instrumented and non-instrumented cultivation systems. Biotechnol. Lett. 29, 703–711. 10.1007/s10529-006-9298-x 17310326

[B40] Myers R. H. Montgomery D. C. Anderson-Cook C. M. (2016). Response surface methodology: process and product optimization using designed experiments. John Wiley and Sons.

[B41] Ojha R. Timin E. Szirovicza L. Xu W. Hepojoki J. (2025). A systematic comparison of in-house prepared transfection reagents in the delivery of mRNA or DNA to a wide range of cultured cells. J. Biol. Chem. 301, 110742. 10.1016/j.jbc.2025.110742 41047124 PMC12603952

[B42] O’Flaherty R. Bergin A. Flampouri E. Mota L. M. Obaidi I. Quigley A. (2020). Mammalian cell culture for production of recombinant proteins: a review of the critical steps in their biomanufacturing. Biotechnol. Adv. 43, 107552. 10.1016/j.biotechadv.2020.107552 32416132

[B43] Promega Corporation (2025). FuGENE® 6 transfection reagent protocol. Available online at: https://www.promega.com/-/media/files/resources/protocols/technical-manuals/101/fugene-6-transfection-reagent-protocol.pdf (Accessed December 10, 2025).

[B44] Rampado R. Peer D. (2023). Design of experiments in the optimization of nanoparticle-based drug delivery systems. J. Control. Release 358, 398–419. 10.1016/j.jconrel.2023.05.001 37164240

[B45] Raymond C. Tom R. Perret S. Moussouami P. L’Abbé D. St-Laurent G. (2011). A simplified polyethylenimine-mediated transfection process for large-scale and high-throughput applications. Methods 55, 44–51. 10.1016/j.ymeth.2011.04.002 21539918

[B46] Reiter T. Brooks P. T. Irber L. Joslin S. E. K. Reid C. M. Scott C. (2021). Streamlining data-intensive biology with workflow systems. Gigascience 10, giaa140. 10.1093/gigascience/giaa140 33438730 PMC8631065

[B47] Rizk S. S. Moustafa D. M. ElBanna S. A. Nour El-Din H. T. Attia A. S. (2024). Nanobodies in the fight against infectious diseases: repurposing nature’s tiny weapons. World J. Microbiol. Biotechnol. 40, 209. 10.1007/s11274-024-03990-4 38771414 PMC11108896

[B48] Salmen W. Hu L. Bok M. Chaimongkol N. Ettayebi K. Sosnovtsev S. V. (2023). A single nanobody neutralizes multiple epochally evolving human noroviruses by modulating capsid plasticity. Nat. Commun. 14, 6516. 10.1038/s41467-023-42146-0 37845211 PMC10579229

[B49] Sharma D. Arora S. Singh J. Layek B. (2021). A review of the tortuous path of nonviral gene delivery and recent progress. Int. J. Biol. Macromol. 183, 2055–2073. 10.1016/j.ijbiomac.2021.05.192 34087309 PMC8266766

[B50] Shen C. Gao Y. Li J. Du H. Liang R. Sun Y. (2020). Intrinsic adjuvanticity of branched polyethylenimine *in vitro* and subcutaneously. ACS Appl. Polym. Mater 2, 1438–1447. 10.1021/acsapm.9b01016

[B51] Tan E. Chin C. S. H. Lim Z. F. S. Ng S. K. (2021). HEK293 cell line as a platform to produce recombinant proteins and viral vectors. Front. Bioeng. Biotechnol. 9, 796991. 10.3389/fbioe.2021.796991 34966729 PMC8711270

[B52] Thompson B. C. Segarra C. R. J. Mozley O. L. Daramola O. Field R. Levison P. R. (2012). Cell line specific control of polyethylenimine‐mediated transient transfection optimized with “Design of experiments” methodology. Biotechnol. Prog. 28, 179–187. 10.1002/btpr.715 22002934

[B53] Tomas-Grau R. H. Ploper D. Ávila C. L. Vera Pingitore E. Maldonado Galdeano C. Chaves S. (2021). Elevated humoral immune response to SARS-CoV-2 at high altitudes revealed by an Anti-RBD “In-House” ELISA. Front. Med. (Lausanne) 8, 720988. 10.3389/fmed.2021.720988 34722566 PMC8551828

[B54] Tzimou K. Catalán-Tatjer D. Nielsen L. K. Lavado-García J. (2024). Unlocking DOE potential by selecting the most appropriate design for rAAV optimization. Mol. Ther. Methods Clin. Dev. 32, 101329. 10.1016/j.omtm.2024.101329 39296857 PMC11406035

[B55] Van M. V. Fujimori T. Bintu L. (2021). Nanobody-mediated control of gene expression and epigenetic memory. Nat. Commun. 12, 537. 10.1038/s41467-020-20757-1 33483487 PMC7822885

[B56] Vega C. G. Garaicoechea L. L. Degiuseppe J. I. Bok M. Rivolta A. A. Piantanida A. P. (2021). ROTADIAL: the first nanobody-based immunoassay to detect group A rotavirus. J. Virol. Methods 298, 114279. 10.1016/j.jviromet.2021.114279 34499967

[B57] Walsh G. Walsh E. (2022). Biopharmaceutical benchmarks 2022. Nat. Biotechnol. 40, 1722–1760. 10.1038/s41587-022-01582-x 36471135 PMC9735008

[B58] Wu Y. (2025). Trends in nanobody technology in industrialization. Discov. Nano 20, 23. 10.1186/s11671-025-04200-2 39928220 PMC11810867

[B59] Yamagata M. Sanes J. R. (2018). Reporter–nanobody fusions (RANbodies) as versatile, small, sensitive immunohistochemical reagents. Proc. Natl. Acad. Sci. 115, 2126–2131. 10.1073/pnas.1722491115 29440485 PMC5834735

[B60] Ye J. Kober V. Tellers M. Naji Z. Salmon P. Markusen J. F. (2009). High‐level protein expression in scalable CHO transient transfection. Biotechnol. Bioeng. 103, 542–551. 10.1002/bit.22265 19199356

[B61] Zhang J. Liu X. Bell A. To R. Baral T. N. Azizi A. (2009). Transient expression and purification of chimeric heavy chain antibodies. Protein Expr. Purif. 65, 77–82. 10.1016/j.pep.2008.10.011 19007889

[B62] Zhao J. Zhu J. Wang Y. Yang M. Zhang Q. Zhang C. (2022). A simple nanobody-based competitive ELISA to detect antibodies against African swine fever virus. Virol. Sin. 37, 922–933. 10.1016/j.virs.2022.09.004 36089216 PMC9797394

[B63] Zylberman V. Sanguineti S. Pontoriero A. V. Higa S. V. Cerutti M. L. Morrone Seijo S. M. (2020). Development of a hyperimmune equine serum therapy for COVID-19 in Argentina. Med. (B Aires) 80 (Suppl. 3), 1–6. 32658841

