# Supplementary material for: A novel PolyAr87-based cell transfection protocol for nanobody expression optimized via a targeted design of transfection approach
Source: Front Bioeng Biotechnol. 2026 Mar 13;13:1720185. doi: 10.3389/fbioe.2025.1720185 (PMC13021770; doi:10.3389/fbioe.2025.1720185)
Supplement: Supplementary file 1 [file DataSheet1.pdf]

Supplementary Material

StdOrder	RunOrder	CenterPt	Block	[DNA] (µg/mL)	[PolyAR] (µg/mL)	Method	Time (min)
**21**	1	1	2	0.5	1	B	5
**28**	2	1	2	3.0	8	A	30
**20**	3	1	2	3.0	8	A	5
**17**	4	1	2	0.5	1	A	5
**29**	5	1	2	0.5	1	B	30
**19**	6	1	2	0.5	8	A	5
**18**	7	1	2	3.0	1	A	5
**32**	8	1	2	3.0	8	B	30
**25**	9	1	2	0.5	1	A	30
**23**	10	1	2	0.5	8	B	5
**24**	11	1	2	3.0	8	B	5
**30**	12	1	2	3.0	1	B	30
**27**	13	1	2	0.5	8	A	30
**26**	14	1	2	3.0	1	A	30
**22**	15	1	2	3.0	1	B	5
**31**	16	1	2	0.5	8	B	30
**11**	17	1	1	0.5	8	A	30
**3**	18	1	1	0.5	8	A	5
**9**	19	1	1	0.5	1	A	30
**12**	20	1	1	3.0	8	A	30
**2**	21	1	1	3.0	1	A	5
**16**	22	1	1	3.0	8	B	30
**14**	23	1	1	3.0	1	B	30
**5**	24	1	1	0.5	1	B	5
**13**	25	1	1	0.5	1	B	30
**1**	26	1	1	0.5	1	A	5
**15**	27	1	1	0.5	8	B	30
**8**	28	1	1	3.0	8	B	5
**4**	29	1	1	3.0	8	A	5
**10**	30	1	1	3.0	1	A	30
**7**	31	1	1	0.5	8	B	5
**6**	32	1	1	3.0	1	B	5

Table S1. Worksheet of the screening two-level full factorial design with all the combinations to test.

StdOrder	RunOrder	PointType	Block	[DNA] (µg/mL)	[PolyAR] (µg/mL)	Time (min)
**15**	1	0	1	1.75	4.5	17.5
**9**	2	2	1	1.75	1.0	5.0
**3**	3	2	1	0.50	8.0	17.5
**2**	4	2	1	3.00	1.0	17.5
**10**	5	2	1	1.75	8.0	5.0
**11**	6	2	1	1.75	1.0	30.0
**13**	7	0	1	1.75	4.5	17.5
**14**	8	0	1	1.75	4.5	17.5
**12**	9	2	1	1.75	8.0	30.0
**5**	10	2	1	0.50	4.5	5.0
**1**	11	2	1	0.50	1.0	17.5
**6**	12	2	1	3.00	4.5	5.0
**4**	13	2	1	3.00	8.0	17.5
**8**	14	2	1	3.00	4.5	30.0
**7**	15	2	1	0.50	4.5	30.0
**30**	16	0	2	1.75	4.5	17.5
**26**	17	2	2	1.75	1.0	30.0
**19**	18	2	2	3.00	8.0	17.5
**16**	19	2	2	0.50	1.0	17.5
**22**	20	2	2	0.50	4.5	30.0
**20**	21	2	2	0.50	4.5	5.0
**29**	22	0	2	1.75	4.5	17.5
**24**	23	2	2	1.75	1.0	5.0
**18**	24	2	2	0.50	8.0	17.5
**25**	25	2	2	1.75	8.0	5.0
**28**	26	0	2	1.75	4.5	17.5
**23**	27	2	2	3.00	4.5	30.0
**21**	28	2	2	3.00	4.5	5.0
**27**	29	2	2	1.75	8.0	30.0
**17**	30	2	2	3.00	1.0	17.5

Table S2. Worksheet of the Box–Behnken design (BBD) with all the combinations to test.

Exp n°	[DNA] (µg/mL)	[PolyAR] (µg/mL)
1	0.75	2.0
2	0.75	5.0
3	0.75	7.5
4	1.75	2.5
5	1.75	5.0
6	1.75	7.5
7	2.75	2.0
8	2.75	5.0
9	2.75	7.5

Table S3. Experimental design matrix for BBD validation.

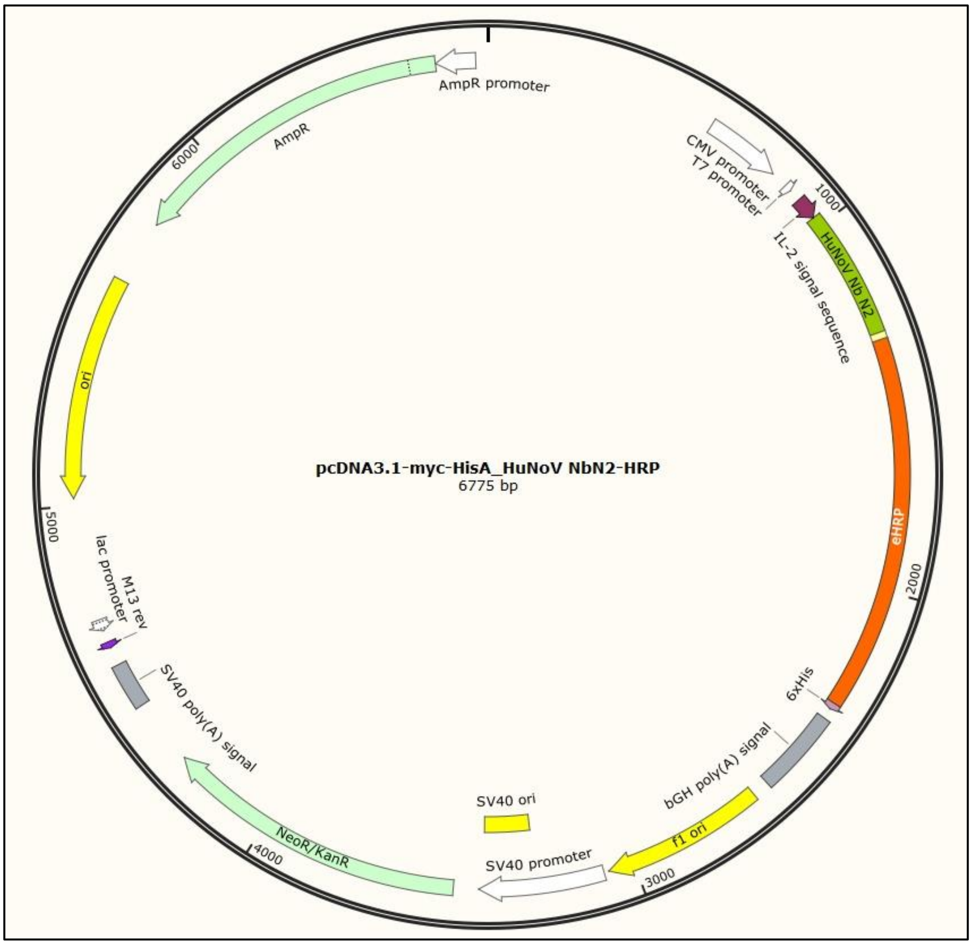

Figure S1

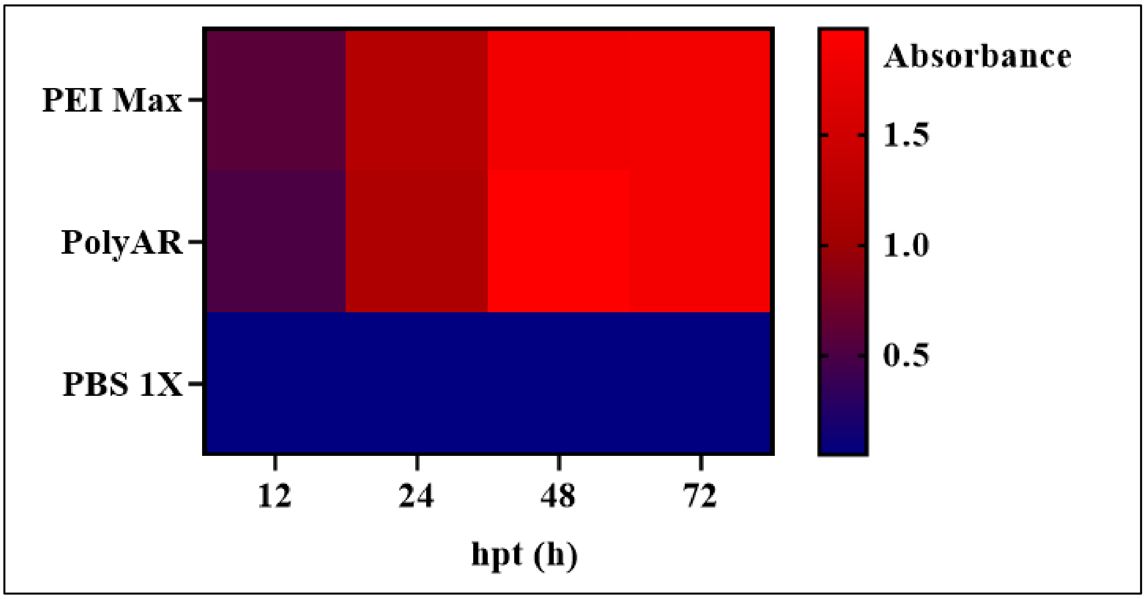

Figure S2

Figure S1. Map scheme of pcDNA3.1 plasmid used for NbN2-HRP expression.

Figure S2. Heatmap of the Expi293F transfection results. Activity measurements were conducted comparing both transfectant PEI Max and PolyAR at different hours post transfection (hpt): 12, 24, 48 and 72 hpt. The experiments were conducted using pcDNA3.1_NbN2-HRP plasmid. After each hpt, the supernatants were separated and mixed with TMB. The absorbances were measured at 450 nm. The absorbance values (averages from duplicates) are expressed on a color scale, with the lower values indicated with blue and the higher values indicated with red.
